# Application and characterization of a novel PVDF-HFP/PVP polymer composite with MoO_3_ nanowires as a protective coating for wood

**DOI:** 10.1038/s41598-023-30622-y

**Published:** 2023-03-01

**Authors:** Jure Žigon, Urška Gradišar Centa, Maja Remškar, Miha Humar

**Affiliations:** 1 Department of Wood Science and Technology, Biotechnical Faculty, University of Ljubljana, Jamnikarjeva ulica 101, 1000 Ljubljana, Slovenia; 2 Center for Experimental Mechanics, Faculty of Mechanical Engineering, University of Ljubljana, Pot za Brdom 104, 1000 Ljubljana, Slovenia; 3 Condensed Matter Physics Department, Jožef Stefan Institute, Jamova 39, 1000 Ljubljana, Slovenia

**Keywords:** Biotechnology, Natural hazards, Risk factors, Engineering, Materials science, Nanoscience and technology

## Abstract

The coatings on wood must sometimes give aesthetic and basic protection to wooden elements and prevent the development and transmission of microorganisms. Several polymers containing different nanoparticles have already been offered to day for this purpose. The research presents a novel poly(vinylidene fluoride-co-hexafluoropropylene) (PVDF-HFP)/polyvinylpyrrolidone (PVP) polymer composite with MoO_3_ nanowires with the ability to form coating films on wood. The films of the developed coating exhibit elastic behaviour, which depends on the coating film thickness [tested wet film thicknesses (90, 180 and 360) µm]. The coating showed the ability to interact well with the surface of common beech (*Fagus sylvatica* L.) wood, in terms of wetting (contact angles of 15.6°), fast spilling on the surface, good penetration of the coating in wood structure and formation of up to 40 µm-thick films with excellent pull-off adhesion strength (6 MPa). An increased roughness of wood coated with C + MoO_3_ was a consequence of wood etching by the dimethylformamide solvent present in the coating. Moreover, the presence of C + MoO_3_ on wood made it considerably more hydrophobic, with contact angle of water raising to 123° from initially 46° measured on uncoated wood. The irradiation of wood surfaces with ultra-violet light resulted in visible colour changes on both uncoated and coated wood. The wood coated with C + MoO_3_ has a good resistance to water, alcohol and dry heat (grade 3 to 4). The antimicrobial testing showed that the presence of MoO_3_ in the coating plays an important role in the resistance of the coated wood to blue-stain fungi and mould development. The developed PVDF-HFP/PVP/MoO_3_ coating has an excellent ability to interact with the wood surface and has the potential to be used as a protection for wood in sensitive environments.

## Introduction

Microorganisms like bacteria, viruses and moulds are present all around us and have colonized all of the habitats in the world, including wood and wooden surfaces. If environmental conditions are suitable, these organisms can remain vital and transmittable for a considerable number of days if not months^1^. The fungal spores in living environment are spread around mostly from one person to another through the air flow and through contact with surfaces, like doors, tabletops, sittings, handles and similar. In general, the growth of moulds usually does not affect the mechanical properties of the wood, but if the wood remains moist long time enough, further development of fungi can cause the formation of soft rot, which can deteriorate the mechanical properties of the wood^2^. Moreover, as reported by Møller and co-workers^3^ and Viitanen and co-workers^4^, the presence of fungi and moulds like *Aspergillus* sp. (e.g. *Aspergillus fumigatus*), *Aureobasidium pullullans*, *Alternaria alternata*, *Cladosporium* sp. (e.g. *Cladosporium herbarum*, *Cladosporium sphaerospermum*), *Mucor* sp., *Penicillium* sp. (e.g. *Penicillium brevicompactum*), *Stachybotrus* sp. (e.g. *Stachybotrus atra*), and *Fusarium solani*, growing on wooden elements installed in living environment, can pose a serious threat for human health. Therefore, the antimicrobial protection of surfaces helps to prevent the spread of infection and diseases caused by microorganisms in the human population^5^.

The susceptibility of wood-based materials for infections with microbes can be determined by monitoring the growth of moulds and fungi on either uncoated or coated surfaces^6^. In general, each coating asset for surface treatment of wooden elements must meet several basic requirements. In order to successfully provide the expected functions of surface protection and a desired aesthetic appearance, the proper interactions between the wooden substrate and the coating are of crucial importance^7^. This way, good wetting of the wooden substrate, sufficient spilling and penetration of the coating in the wood structure defines the mechanical anchoring and adhesion of the coating film to the substrate. At the same time, the physical performance and resistance properties of the formed surface system are indicated by the surface hydrophobicity, resistance to cold liquids and changes in the surface appearance caused by the irradiation of ultra-violet (UV) light.

In the last decades, the trends in the development of new coating systems for wood are focused on searching and investigating the coating formulations with new binders and nanoparticles (e.g. TiO_2_, SiO_2_, ZnO, Al_2_O_3_, nanocellulose, graphene) as additives for improvement of various mechanical and physical properties^8^, as well as for preservation purposes^9^. Moreover, the need for effective and cost-affordable fungicidal agents (biocides) with long-acting and without risk to human health and the environment is extremely high.

Molybdenum trioxide (MoO_3_) was reported as one of the promising antimicrobial active substances^10,11^. In contrast to some other fungicidal compounds, the health risks in the case of MoO_3_ are minimal, as Mo is one of the essential elements in trace amounts and is a cofactor for the formation of enzymes in human bodies^12^. Otherwise, the 1 h-exposure of human keratinocyte cells (HaCaT) to MoO_3_ nanowires in concentration up to 1 mg × mL^–1^ after does not show any effect on cell survival^13^.

Minubaeva^14^ attributed the antimicrobial efficacy of MoO_3_ to its dissolution in water and the formation of antimicrobial molybdic acid (H_2_MoO_4_) (1):

1 $${\text{MoO}}_{{3}} {\text{ + H}}_{{2}} {\text{O = }}\left[ {{\text{HMoO}}_{{4}} } \right]^{-} {\text{ + H}}^{ + } { } \leftrightarrow {\text{ H}}_{{2}} {\text{MoO}}_{{4}} { }$$

Then, the H_2_MoO_4_ dissolves also into oxonium (H_3_O^+^) and molybdate (${\text{MoO}}_{4}^{2 - }$) ions, as follows (2):

2 $${\text{H}}_{{2}} {\text{MoO}}_{{4}} {\text{ + 2H}}_{{2}} {\text{O }} \leftrightarrow {\text{ 2H}}_{{3}} {\text{O}}^{ + } {\text{ + MoO}}_{{4}}^{{{\text{2{-}}}}} ,$$

while the protonation of the ${\text{MoO}}_{4}^{2 - }$ ions takes place simultaneously.

In principle, the diffusion of H_3_O^+^ ions through the cell wall of microbes causes an imbalance in pH value and the cell's enzyme and transport system. Therefore, for the antimicrobial action of MoO_3_ nanoparticles on the surface, the rate of MoO_3_ solubility in water (brought on the surface by microbes, air humidity, or cleaning) is of a critical in this process. Because that antimicrobial action is triggered by the presence of water in the liquid or vapour phase, this type of MoO_3_-containing nanocomposite can be an environmentally friendly sustainable solution because the use of detergents and disinfectants can be reduced.

The incorporation of MoO_3_ into polymeric binder allows the application of such polymer as a coating on various solid substrates, with controlled release rates and long-term use. Shafaei and co-authors^15^ reported the effect of different MoO_3_ crystal structures of micrometer size embedded in different polymer composites on antimicrobial activity against the bacteria *S. aureus*, *E. coli* and *P. aeruginosa*. The authors concluded that direct contact between the bacteria and MoO_3_ is needed that the H_3_O^+^ ions can diffuse through the bacterial membrane. All tests so far have been done with commercially available MoO_3_ with a micrometer size. Because the rate of solubility and consequent onset of antimicrobial activity depend on the specific surface area of MoO_3_, the nanoparticles (i.e. nanowires and nanotubes) of MoO_3_ have an advantage over the materials with MoO_3_ of micrometer size. In this manner, the high antimicrobial efficacy of MoO_3_ nanowires incorporated into poly(vinylidene fluoride-co-hexafluoropropylene) (PVDF-HFP) and water-soluble polyvinylpyrrolidone (PVP) polymers was proven in our recent studies^16,17^.

Several examples of the use of coatings containing nanoparticles with antimicrobial properties well illustrate the potential use of such coating on furniture surfaces^18,19^. The aim of this research was to introduce the MoO_3_ nanoparticles into PVDF-HFP/PVP polymer composite acting as a binder to obtain a completely new type of coating for wood with improved physical properties and antimicrobial functionalities. Firstly, the basic properties (solids content and surface tension) of the synthetized liquid PVDF-HFP/PVP/MoO_3_ composite coating were determined. As the coating film thickness greatly influences the properties of the coating films and the properties of the surface systems, the coating was applied in three different wet-film thickness, namely 90 µm, 180 µm and 360 µm. The relation between the coating film thickness and its hardness was determined with a pendulum damping test. The wettability of wood with the coating was studied with contact angle (CA) measurements and by monitoring the spilling of the coating droplets over the surface after application on wood. After application of the coating on the surface of a wood, several properties of the coated wood were determined: Microstructure and surface roughness, hydrophobicity, stability against UV light irradiation, resistance properties, and the coating adhesion. Finally, the ability of the developed coating to prevent the growth of blue-stain fungi and moulds on the wood was tested.

## Materials and methods

### Synthesis of PVDF-HFP/PVP/MoO_3_ composite coating

Up to 3 μm-long MoO_3_ nanowires, with a diameter ranging from 100 to 150 nm, were obtained from Mo_6_S_2_I_8_ nanowires (Nanotul d.o.o., Ljubljana, Slovenia). These were synthesized in 236 h under chemical transport reaction in evacuated (104 Pa) quartz ampules and heated (800 °C) from molybdenum powder, sulfur powder and iodine beads (molar ratio 6:2:11.6). By oxidation at 285 °C for 24 h^20^ the MoO_3_ nanowires were grown in the orthorhombic crystal structure. Their solubility in pure water is (2.03 ± 0.09) mg × ml^‒1^ and the solutions reach the pH value of 3.8, as shown in a recent study^17^.

The polymer blend (C–MoO_3_) was synthesized by separately dissolving and mixing (120 min, 80 °C, 400 min^–1^) PVDF-HFP (Sigma-Aldrich, St. Louis, Missouri, USA) and PVP K30 (Sigma-Aldrich) in dimethylformamide (DMF, CARLO ERBA Reagents S.A.S., Milan, Italy). Then solutions of the polymers were mixed together and mixed for another 120 min. For the formation of nanocomposite, the MoO_3_ nanowires were dispersed in PVP solution and mixed for 120 min. Finally, the PVDF-HFP, PVP and MoO_3_ were mixed with a corresponding ratio 69:23:8 for another 120 min and the final coating product (C + MoO_3_) was obtained. As previously reported^16^, the C + MoO_3_ polymerizes and forms a solid film after drying for 120 min in a hot (80 °C) air atmosphere.

### Determination of liquid coating properties

For the determination of solids content, a total of (1 ± 0.1) g of the liquid C + MoO_3_ was applied in three 50 mm diameter Petri dish and dried at a temperature of 125 °C for 60 min. The solids content (in %) was calculated from the initial C + MoO_3_ mass and its mass after drying, as prescribed by the standard EN ISO 3251^21^.

The surface tension of the liquid C + MoO_3_ was determined according to Du Noüy ring method^22^ on processor tensiometer K100 (Krüss, Hamburg, Germany) using the corresponding software (Krüss LabDesk, Krüss). Five measurements were performed.

### Determination of liquid coating properties

The coating films with wet thicknesses of 90 µm, 180 µm and 360 µm were created on the glass plates of 100 mm × 100 mm (3 plates per each film thickness) with a manual quadruple film applicator (Model 360, Erichsen GmbH & Co. KG, Hemer, Germany). The coating hardness was determined with pendulum-damped method^23^ using the König pendulum damping tester model 299/300 (Erichsen GmbH & Co. KG). The glass plates with applied coatings were placed for 120 min in an oven for drying (80 ± 2) °C and conditioned at (23 ± 2) °C and air relative humidity of (30 ± 5)% for 21 days afterwards. The coating hardness corresponded to the damping time of the pendulum swinging on the coating surface from 6° to 3° with respect to the normal axis (five measurements per sample). The longer the damping time, the harder the coating film.

### Preparation of wood samples

**Figure 1:**
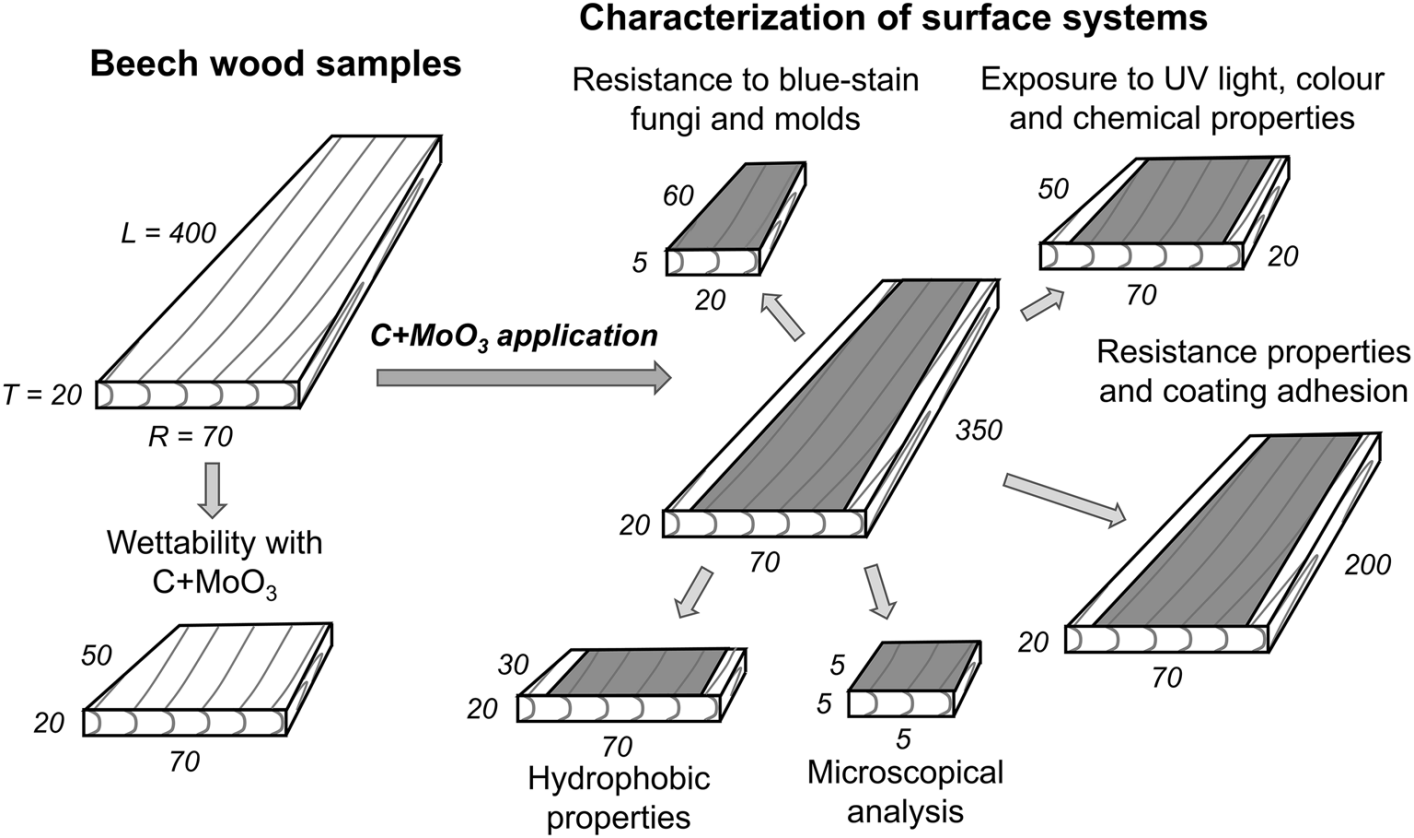
Distribution of wood samples preparation for different studies. The numbers present the sample size in individual wood grain direction (in millimeters): L—longitudinal, R—radial and T—tangential.

The quartersawn common beech (*Fagus sylvatica* L.) sapwood planks, stored for two years in a room with air relative humidity of 30% and temperature 23 °C, were selected as a studied wooden substrate material. The gravimetrically determined nominal density of wood with (7.1 ± 0.4)% moisture content was (688.7 ± 24.5) kg × m^–3^. The wood was mechanically processed by sawing and peripheral planing into eight samples with dimensions (400 mm × 70 mm × 20 mm) and corresponding wood grain orientation longitudinal × radial × tangential (L × R × T). The samples were furtherly divided to the smaller samples for each analysis, as schematically presented in Fig. 1. All the analyses were conducted on the surfaces with R wood grain orientation, which were manually sanded with rotational-vibrational sanding machine (paper grit P180).

### Determination of wood wettability with coating

The wetting properties of wood surface with coating were estimated on the basis of the behavior of coating droplets after application on the surfaces of 3 uncoated samples (L = 50 mm). Five 5 µL-droplets of coating were applied on each sample with optical goniometer Theta (Biolin Scientific Oy, Espoo, Finland). The camera of goniometer enabled to record the motion of the droplet and to measure the CA of droplet following the Young–Laplace analysis^24^ continuously during first 60 s after application. The final CA was measured 2 h after droplet application.

After application on wood, the spilling of the coating droplets was determined by applying five 20 μL-droplets of coating from 20 mm above the sample surface using a pipette fixed on the stander. The spilling of the droplets 1, 2, 4, 6, 8, 10, 15, 20, 25, 30, 45, 60 and 7200 s after the application was recorded from above with a photo camera (D5600, Nikon, Tokyo, Japan). The photos were analyzed by measuring the spilling area (in mm^2^) of the droplets using the Fiji software (ImageJ 1.46d, Bethesda, Maryland, USA)^25^.

### Formation of surface systems on wood

The surface systems on wood were formed by applying the coating with wet thicknesses of 90 µm, 180 µm and 360 µm with a manual quadruple film applicator (Erichsen GmbH & Co. KG) on two wood samples (L = 350 mm) for each film thickness. The coated wood samples were dried for 120 min in an oven at (80 ± 2) °C and afterwards conditioned at (23 ± 2) °C and air relative humidity of (30 ± 5)% for 7 days before further analyses.

#### Microscopic investigation of coated wood and determination of surface roughness

The surface roughness of the uncoated and coated samples was examined on five selected spots on each sample with a LEXT OLS5000 confocal laser scanning microscope (CLSM, Olympus, Tokyo, Japan). The analyses at 5 × magnification provided the scanned area of (2560 × 2560) μm^2^. The microscope was equipped with a 405 nm laser light source with a maximum lateral resolution of 0.12 μm. The corresponding software (OLS50-S-AA, Olympus) was used to calculate the arithmetical mean height of the surfaces *S*_a_^26^.

A droplet of C + MoO_3_ was deposited on an aluminum plate of (10 × 10 × 1) mm^3^ and polymerized. Moreover, two samples of about (5 × 5 × 5) mm^3^ were cut out of each coated wood sample. The microscopic structures of the cured C + MoO_3_ droplet, coated wood surfaces and their cross-sections were analyzed with a scanning electron microscope Quanta 250 (Thermo Fisher Scientific, Hillsboro, USA). A gold conductive layer was sputtered on the samples prior to observations. The images were acquired with the Everhart–Thornley detector in high vacuum (0.078 Pa) with an electron source voltage of 5.0 kV, at a working distance of 10 mm, a spot size of 3.0 nm, and by the time of electron beam passage through the sample of 45 μs.

#### Determination of coated wood hydrophobic properties

In order to detect the hydrophobic characteristics of wood coated with coating films of different thicknesses, again the optical goniometer Theta (Biolin Scientific Oy) was used. Ten 5 µL-droplets of demineralized water were applied on the samples (L = 30 mm) coated with each film thickness and the formed CAs were monitored continuously for 60 s.

#### Exposure of coated wood to UV light and monitoring of visual and chemical properties

The coated wood samples were exposed to UV light originating from 450 mm-distant Ultra-Vitalux 300 W bulb (Osram, Munich, Germany) for 96 h. The colour changes of the samples during exposure to UV light were monitored after 3, 6, 24, 48, 72, and 96 h by measuring L* (0 = black, 100 = white), a* (negative = green, positive = red) and b* (negative = blue, positive = yellow) colour space components using the spectrophotometer SP62 (X-Rite, Grand Rapids, USA) with the D65 light type. Colour changes of the sample surfaces after the certain time of exposure to UV (ΔE*) were calculated according to Eq. (3):

3 $$\Delta E^{*} = \sqrt {\left( {L^{*}_{n} - L^{*}_{0} } \right)^{2} + \left( {a^{*}_{n} - a^{*}_{0} } \right)^{2} + \left( {b^{*}_{n} - b^{*}_{0} } \right)^{2} } ,$$

where *n* present the parameter value after a certain interval of exposure to UV and *0* present the initial value of a certain parameter. Meanwhile, the photodegradation of the uncoated and coated samples due to UV light irradiation was quantified with attenuated total reflection Fourier transform infra-red (ATR-FTIR) spectrometer Spectrum Two (PerkinElmer Inc., Waltham, USA). The spectra were measured in 3 spots (16 scans per spot) on individual sample, in a wavelength region from 600 to 4000 cm^–1^ at a resolution of 0.5 cm^–1^. The relevant absorption bands were interpreted with the Spectrum software (version 10.5.3, PerkinElmer Inc.).

#### Determination of resistance properties of coated wood

The resistance of the surface to cold liquids was determined according to the method prescribed by EN 12720^27^. Soft filter paper disks (diameter 25 mm, grammage 450 g × m^–2^) were immersed for 30 s in deionized water and water solution (48%) of ethanol^28^. Three disks soaked into each liquid were placed on the surface of the coated samples, immediately covered with a Petri dish and left as so for 1 h. After the exposure period, the disks were removed, and the remainder of the liquids was carefully wiped off with a soft paper towel.

In order to determine the resistance of the surface systems to dry heat^29^ the aluminum disks were heated to 85 °C and 200 °C and placed on the surfaces of the coated samples for 20 min and removed afterwards.

After 24 h, all the tested surfaces were cleaned, examined for damage in a standardized viewing cabinet and rated according to a numerical evaluation code from 5 to 1 (5—no change, 1—severe change).

#### Determination of coating adhesion to wood

For determination of coating adhesion ten aluminum dollies were bonded to each coated surface using an epoxy adhesive (Endfest 300, UHU, Bühl, Germany). After 24 h, the cured epoxy adhesive around the perimeter of each dolly was cut to the substrate and the dollies were pulled-off at a tensile stress rate of 1 MPa × s^–1^ using a PosiTest AT pull-off adhesion tester (DeFelsco Corporation, Ogdensburg, USA)^30^. Besides the breaking strength, the fractures between the dollies and the surface of coated wood were visually assessed.

### Blue-stain fungal test and resistance to moulds

The blue-stain fungal test was performed according to modified standard EN 152-1^31^, by exposing the samples coated with C + MoO_3_, C–MoO_3_ and uncoated samples (10 samples of each series) to blue-stain fungi *Aureobasidium pullulans* (de Barry) Arnaud (ZIM L060) and *Sclerophoma pithyophila* (Corda) Hohn (ZIM L070) for the period of 8 weeks. The specimens were submerged to spore suspension and afterwards transferred to experimental jars (250 mL) with 15 mL of spore suspension. After the termination of the exposure period, the evaluation of surface blue-staining on each specimen was visually rated on a scale of 0–3 with the naked eye and light microscope, using the criteria prescribed in standard^31^ (Table 1).

**Table 1:** Ranking system for determination of the resistance to blue-stain fungi and moulds.

Blue-stain fungal test^31^		Resistance to moulds^33^	
Rating	Description	Rating	Description
0	Not blue stained	0	No mould growth
1	Weakly blue-stained; small spots less than 2 mm in diameter	1	Initial growth, one or a few hyphae and no conidiophores
2	Moderately blue-stained; up to one-third of the surface is blue-stained	2	Sparse but clearly established growth; often conidiophores are beginning to develop
3	Severely blue-stained; more than one-third of the surface is blue-stained	3	Patchy, heavy growth with many well-developed conidiophores
		4	Heavy growth over more or less the entire surface

The resistance of samples to mould overgrowth was assessed according to a modified American wood protection association standard E24-12^32^. The test took place in a moulds box with a controlled temperature of 25 °C and relative air humidity of (95–98)% for 8 weeks. Five samples of (60 × 20 × 5) mm (L × R × T) from each series were placed on a mesh rack above the water with moist soil with treated surfaces facing the upside. The soil was inoculated with a suspension of spores and mycelium of *Aureobasidium pullulans* (d. By.) Arnaud, *Aspergillus niger* Van Tiegh, *Alternaria tenuissima* (Kunze) Wiltshire, and *Penicillium citrinum* Thom. Mould coverage of the samples was monitored by scanning the samples weekly. Mould growth on each specimen was visually rated on a scale of 0–4 with the naked eye and light microscope, using the criteria proposed by Johansson and co-workers^33^.

## Results and discussion

### Liquid coating properties

The non-volatile content in the C + MoO_3_ was (21 ± 2)%. The surface tension of the liquid C + MoO_3_ with a temperature of 23 °C was (33.22 ± 0.04) mN × m^–1^, while heating the liquid C + MoO_3_ to 80 °C decreased its surface tension to (29.87 ± 0.07) mN × m^–1^.

### Coating film hardness

**Figure 2:**
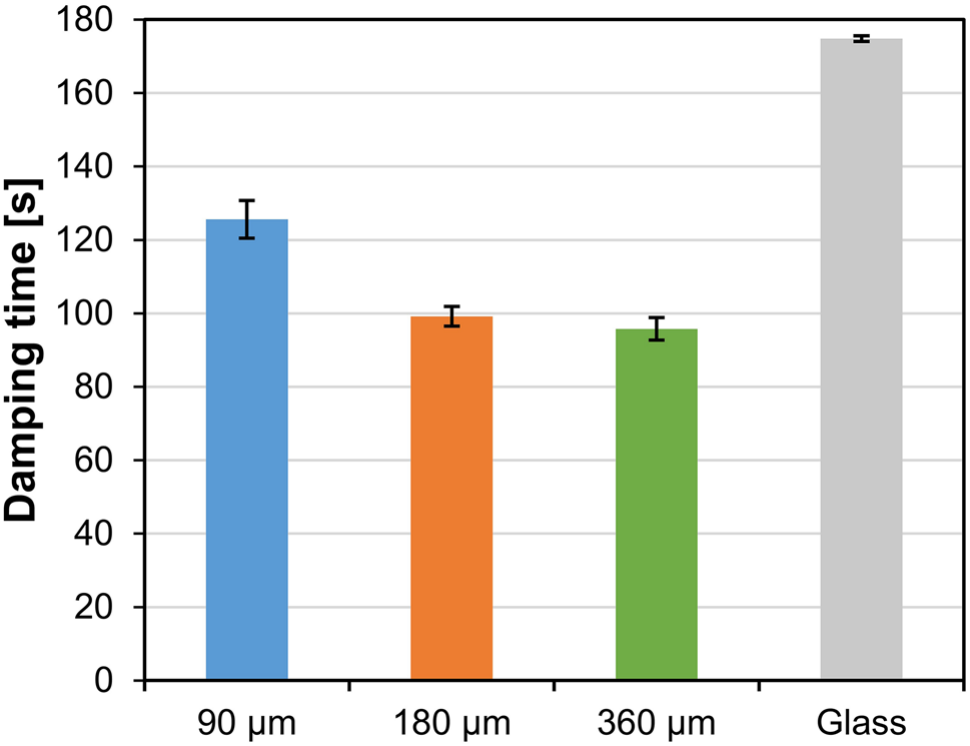
Damping times determined with pendulum hardness tester on a glass with applied C + MoO_3_ films with different wet film thicknesses and on uncoated glass.

In the pendulum test for measuring of hardness of a coating, amplitude of oscillation is measured as a function of time. In general, the hardness of a coating is greatly related to its thickness; the harder is the analyzed coating film, the greater is the oscillation time of the pendulum^34^. Damping of the oscillations is also affected by elastic deformations present in a coating film. The damping time on the uncoated glass was (175 ± 1) s. Among the C + MoO_3_ films, the longest damping time (126 ± 5) s, i.e. the smallest damping effect, was measured on the 90 µm-thick film (Fig. 2). With the increase of the film thickness to 180 µm, the damping time shortened for 21% to (99 ± 3) s. However, on the thickest C + MoO_3_ film (360 µm) the damping time shortened only slightly (3.5%) to (96 ± 3) s. The results show that the C + MoO_3_ films exhibit elastic behavior, because their hardness is not linearly correlated with the coating film thickness. The damping times were comparable to the damping times measured on films of indoor wood coatings^35^, but notably shorter than on films of exterior wood coatings^36^.

### Wettability of wood with a coating

**Figure 3:**
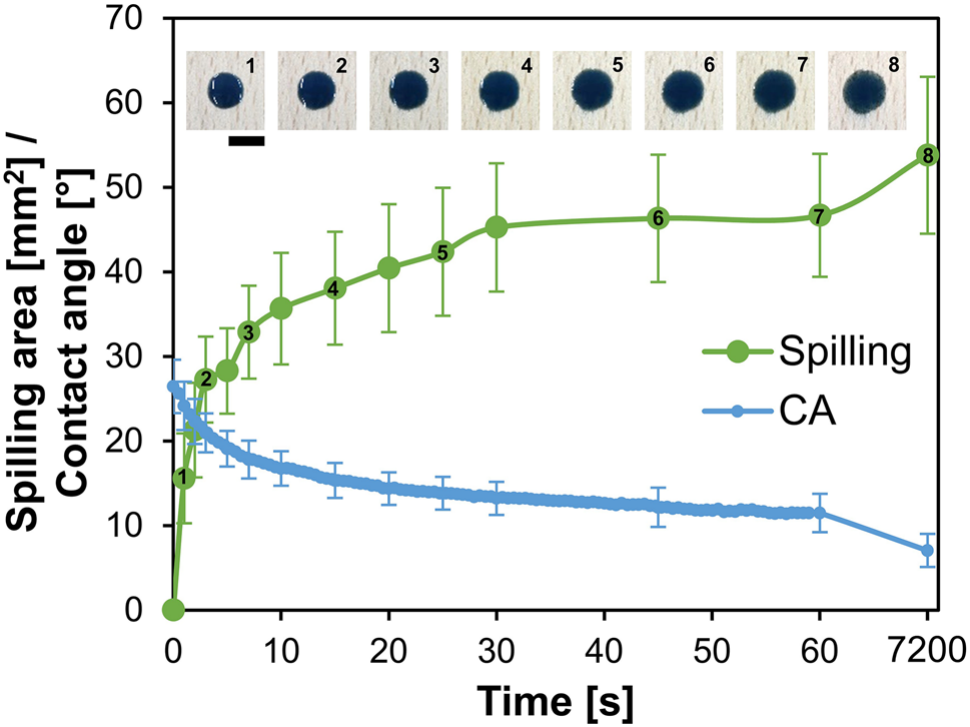
Evolution of the spilling area and contact angle of C + MoO_3_ droplets applied on beech wood. The photos are showing the top-view photos of the applied droplets after correspondent time after deposition. The length of the scale bar is 5 mm.

The evolution of CAs of C + MoO_3_ droplets and spilling areas after application of the coatings on beech wood substrate are shown in Fig. 3. The C + MoO_3_ expressed good wetting abilities on the surface of the wood. In average, the initial detected CA was (26.4 ± 3.2)°. With time, it gradually decreased to (11.5 ± 3.2)° in 60 s of measurements. After 120 min, the CA of the droplets decreased to (7.1 ± 3.2)° and completely wetted the surface of wood.

The spilling area of the C + MoO_3_ 1 s after droplet deposition on the surface of wood was (15.6 ± 5.3) mm^2^ and rapidly increased to (28.3 ± 5.0) mm^2^ in the first 5 s after deposition. After 60 s and 120 min of observations, the spillings of C + MoO_3_ were (46.7 ± 7.3) mm^2^ and (53.8 ± 9.3) mm^2^, respectively.

### Surface systems properties

#### Microstructure and surface roughness of coated wood

**Figure 4:**
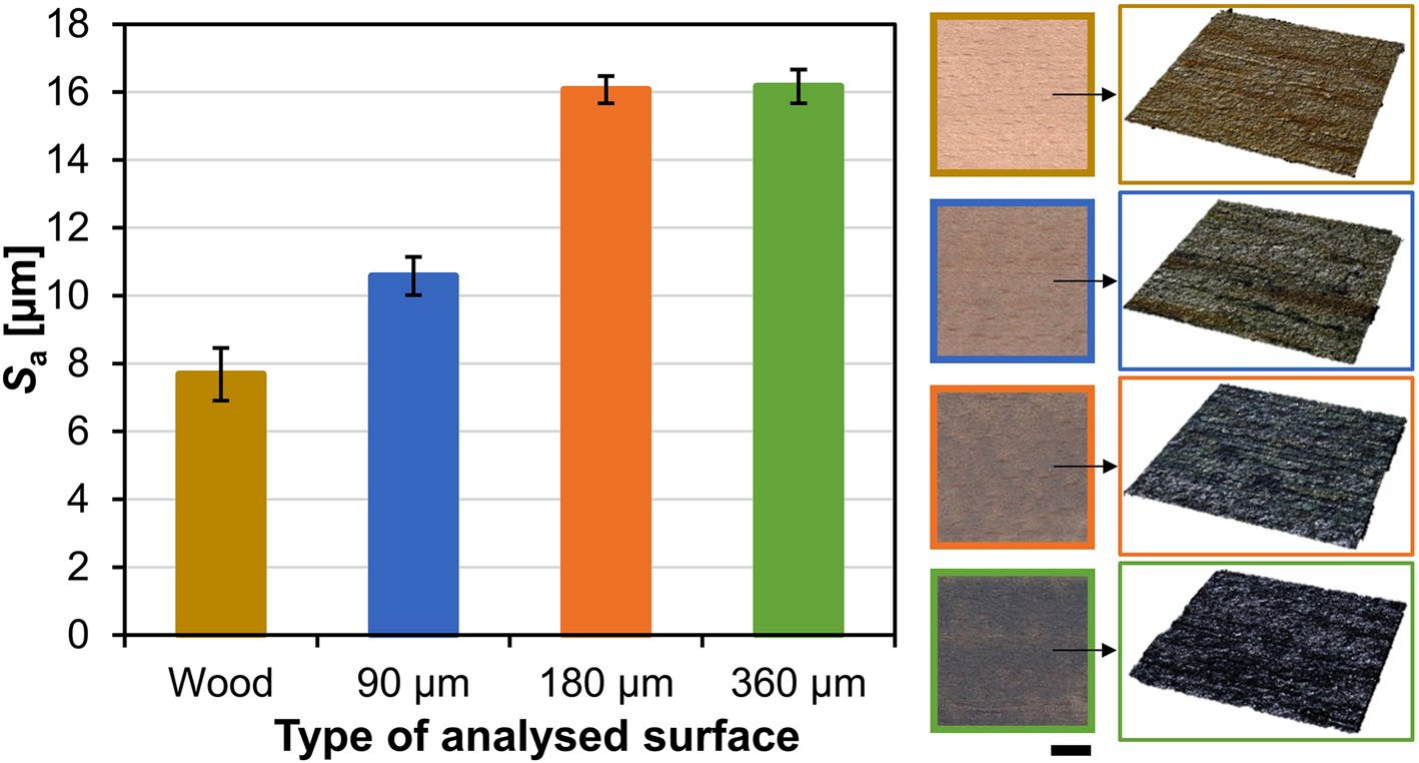
Chart shows the arithmetic mean surface roughness of uncoated wood and wood coated with different thicknesses of C + MoO_3_ wet films. The images on the right show the appearance of the samples surface: left column show the photos of the surfaces (length of the scale bar is 10 mm), right column show the 3D images of each type of analyzed surface as obtained with CLSM (analyzed surface of (2560 × 2560) μm^2^).

The images and measured arithmetic mean surface roughness of uncoated (sanded) wood and wood coated with different thicknesses of wet films of C + MoO_3_ are shown in the Fig. 4. The coated surfaces of wood were rougher than uncoated surfaces of wood. Moreover, the roughness of coated wood surface increased with the larger thickness of C + MoO_3_ film. The application of the thinnest (90 µm) C + MoO_3_ film increased the roughness of samples from (7.7 ± 0.8) µm to (10.6 ± 0.6) µm, or for about 38%. The application of thicker (180 µm) C + MoO_3_ film increased the roughness of wood even more (to (16.0 ± 0.4) µm), that is about 110% increase. As reported in different studies, the dimethylformamide, which was used in our coating, as an ionic liquid, can dissolve cellulose^37^, hemicelluloses^38^ and lignin^39^ in wood. Therefore, the increasing surface roughness with increasing coating film thickness is most probably related with the greater content of dimethylformamide solvent in the C + MoO_3_ film, which caused the etching of the underlying wood surface. However, the application of the thickest (360 µm) coating film did not increase the surface roughness substantially any further ((16.2 ± 0.5) µm). In this case, despite the etching of wood surface by the dimethylformamide solvent, the content of the remaining polymer blend on the wood surface seemed to be thick enough to hinder the etching effect.

**Figure 5:**
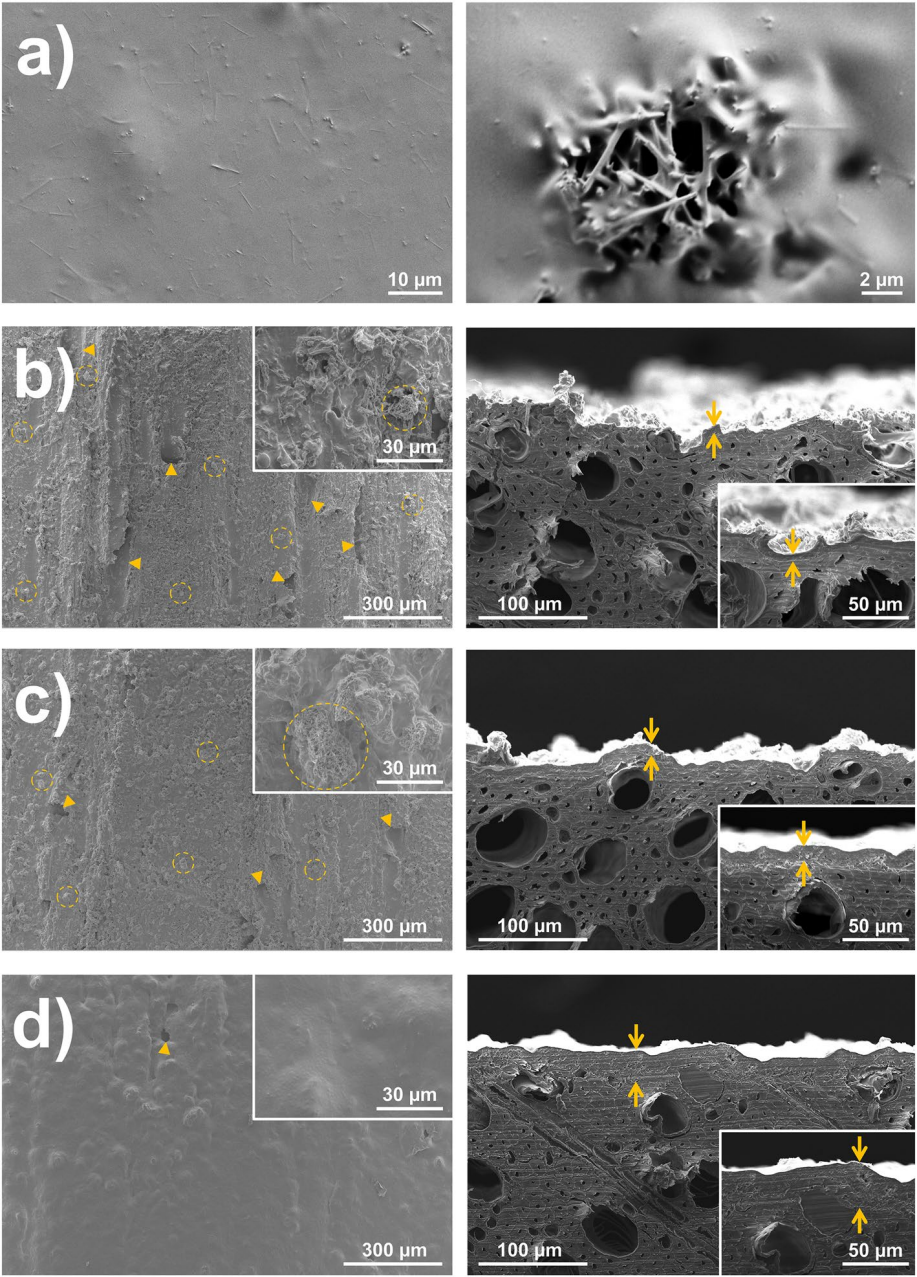
SEM images in (**a**) show the microstructure of the cured C + MoO_3_ droplet (left) and the MoO_3_ nanowires embedded in the PVDF-HFP/PVP polymer (right). Images (**b**)–(**d**) show the surface of wood coated with different wet film thicknesses of C + MoO_3_: (**b**) 90 µm, (**c**) 180 µm and (**d**) 360 µm. The left images show the surfaces structure: Triangular arrows are pointing the pores in the coating films, while the clusters of MoO_3_ nanowires are encircled with the dashed lines. The right images show the cross-sections structure: The clear transition between cellular wooden substrate (bottom) and the film of C + MoO_3_ is clearly visible only in (**c**) and (**d**). The estimated dry film thicknesses present the distance between the contradictory arrows.

Images in Fig. 5a show the microstructure of the cured C + MoO_3_ droplet. A more detailed analysis of the coated wood samples with SEM revealed the microstructure of the samples' surface and the microstructure of the samples' cross-sections with the properties of the interface between wood and C + MoO_3_ film (Fig. 5b‒d). The analysis revealed the presence of MoO_3_ nanowire clusters embedded in the polymer over the entire surface of the coated samples. The surface porosity of the wood beech substrate is reflected in the uneven and porous film of C + MoO_3_. The thicker the coating film was, the smaller pores with a diameter of up to few tens of µm were on the surface. On the sample coated with the thickest (360 µm) C + MoO_3_ film, clusters of MoO_3_ nanowires were no longer detected.

The thicknesses of the dry coating films were estimated on the cross-section images of the coated samples. The dry C + MoO_3_ film thickness on sample coated with wet film thickness of 90 µm varied from about (3 to 8) µm, on sample coated with 180 µm-thick film from about (10 to 16) µm and on sample coated with 360 µm-thick film from about (15 to 40) µm.

#### Hydrophobicity of coated wood

**Figure 6:**
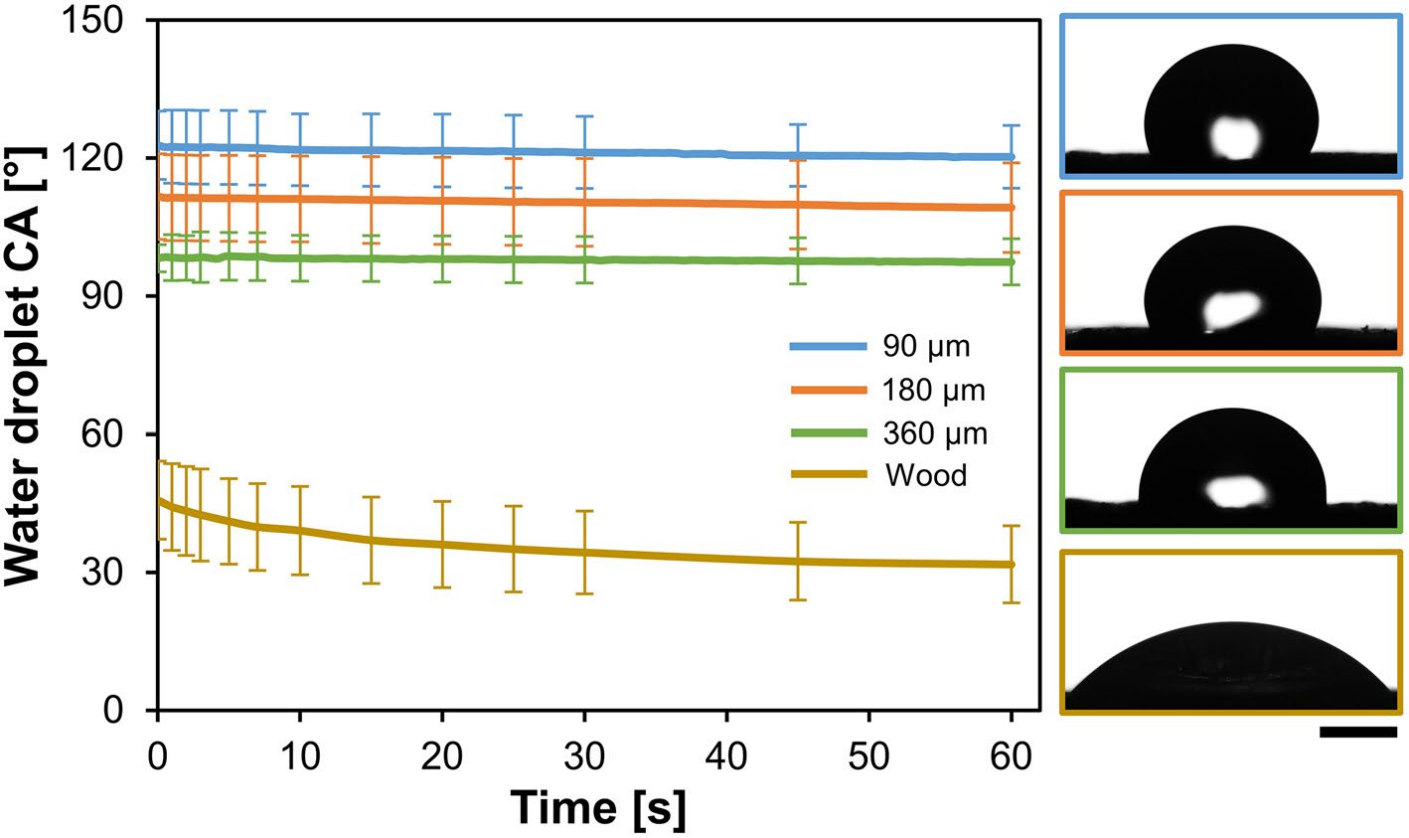
Evolution of water droplets CAs after deposition on the uncoated beech wood surface and on surfaces coated with C + MoO_3_ films different thickness. The images on the right show the photos of the water droplets acquired 2 s after deposition on each type of surface. The length of the scale bar is 1 mm.

The behavior of water droplets deposited on coated wood was a good indicator of the ability of the C + MoO_3_ film of particular thickness to protect the wooden substrate from water. The uncoated wood surface showed a notably higher affinity to absorb water droplets than the wood surface coated with C + MoO_3_ films (Fig. 6). After deposition on uncoated wood, the water droplets formed the CA with the wood surface of (45.7 ± 8.5)°. In 60 s of monitoring, the CA decreased to (31.7 ± 8.4)° due to the penetration of water into the substrate and spreading over the wood surface. The wood surface coated with C + MoO_3_ exhibited hydrophobic properties, which depended on the C + MoO_3_ film thickness. The developed CAs of water decreased only by about 1° during the 60 s measurements, regardless of C + MoO_3_ film thickness. The wood surface coated with the thinnest film of C + MoO_3_ exhibited the most hydrophobic character with initial water CA of (122.8 ± 7.4)°. The initial CAs of water droplets on surfaces of wood coated with 180 µm and 360 µm of C + MoO_3_ were lower (111.6 ± 9.3 and 98.2 ± 2.9)°, respectively. These surfaces can still be denoted as hydrophobic^40^. This brings to the conclusion that the thinner is C + MoO_3_ film, the higher is the hydrophobic character of the coated wood surface. The reason for this lays in the influence of C + MoO_3_ film thickness on the surface roughness. The larger amount of C + MoO_3_ on wood leads to increased surface roughness (etching of wood) and to lower developed CAs of water droplets. The relation between the higher surface roughness of poly-dimethylsiloxane and lower water CAs was, as for example, reported by Juárez-Moreno and co-authors^41^.

#### Changes caused by the UV light irradiation

##### Changes of colour

**Figure 7:**
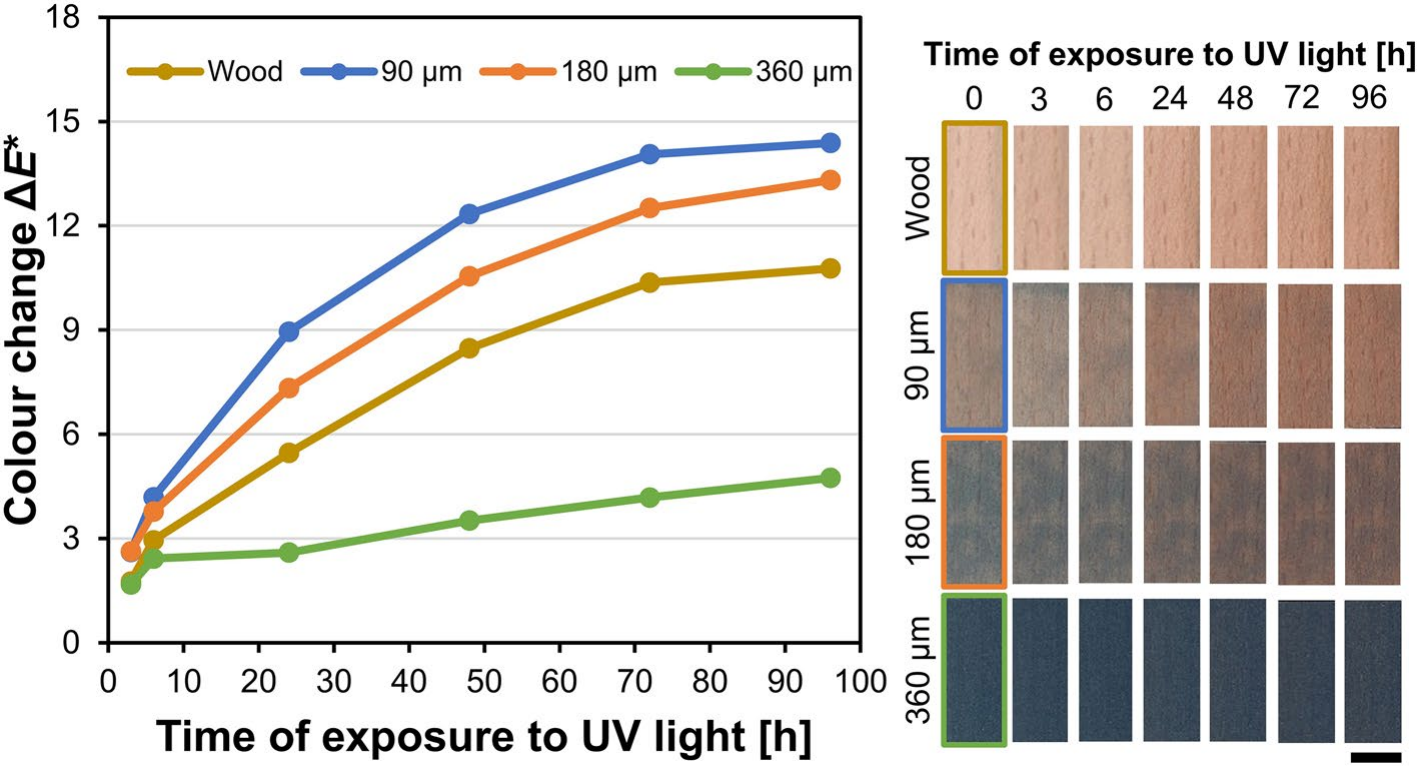
Colour changes of uncoated and coated wood samples during 96 h of irradiation with UV light. The length of the scale bar is 10 mm.

The irradiation of the coated wood samples with UV light caused the changes in the colour of the samples shown in the Fig. 7. On all samples, the most intensive changes in colour occurred during the first 6 h of exposure, and then to the final 96 h, the change rates gradually slowed down. The uncoated wood samples became darker (ΔL* = – 8.7), more reddish (Δa* = 5.0) and yellower (Δb* = 3.8), signalizing the structural changes in lignin^42^. Since the C + MoO_3_ films were semi-transparent, the observed colour changes on the coated samples resulted from combined changes that took place in the wood surface and coating films. After 96 h of exposure to UV light, the largest changes (ΔE* = 14.4) were observed in samples coated with 90 µm-thick coating film, followed by the samples coated with 180 µm-thick coating film (ΔE* = 13.3) and uncoated wood (ΔE* = 10.8). The colour changes of samples coated with 360 µm-thick coating film were lower (ΔE* = 4.8), but still intensive enough to be detected with the naked eye (ΔE* ≥ 2)^43^.

### ATR-FTIR spectra

**Figure 8:**
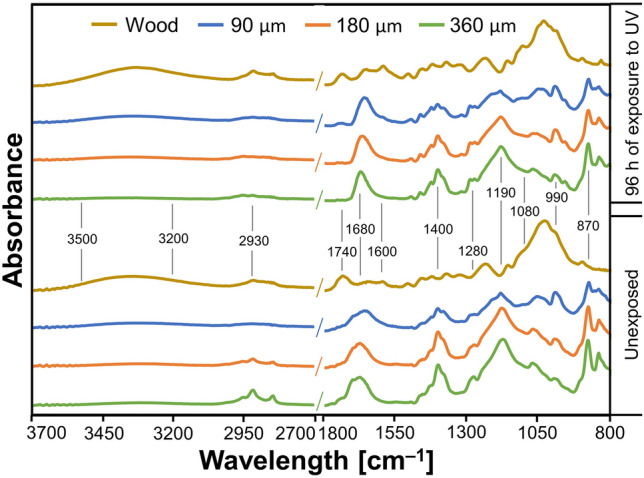
ATR-FTIR spectra of wood and coated samples in the wavelength region between (800 and 1800) cm^‒1^ and region between (2700 and 3700) cm^‒1^, recorded before (unexposed) and after 96 h of exposure to UV light.

The spectra obtained with ATR-FTIR spectrometer on the uncoated wood surface and wood surface coated with C + MoO_3_ of different film thicknesses before and after exposure to UV light are shown in Fig. 8.

The C + MoO_3_ films hindered the chemical composition of uncoated wood, regardless of film thickness. By increasing the C + MoO_3_ film thickness, the broad peak of hydroxyl (OH) groups between (3500 and 3200) cm^–1^ decreased. The peaks at 2930 cm^–1^ (signalizing asymmetric CH_2_ stretching) and 2840 cm^–1^ (signalizing symmetric CH_2_ stretching) intensified with increased C + MoO_3_ film thickness. These peaks originate from crystalline cellulose in wood^44^ and identify the dissolution of wood tissue by C + MoO_3_. The peak at 1740 cm^–1^ typical for C=O stretch in hemicelluloses (xylan)^45^, completely disappeared, which is another indicator of wood dissolvement by C + MoO_3_. On the other hand, a new main peak between (1660 and 1680) cm^–1^, with a shoulder peak at 1700 cm^–1^ (attributed to C=O stretching of PVP) appeared^46^. The shift of the main peak from 1660 cm^–1^ to 1670 cm^–1^and 1680 cm^–1^ with higher C + MoO_3_ thickness (from 90 to 180 and 360) µm is related to the intermolecular interaction between the polymers^47,48^. The peak at 1670 cm^‒1^ was present due to C=O bound by PVP–PVP dipole interactions or having weak hydrogen bonding to another species. In the case of C=O groups strongly bound to another species, the peak was more shifted to 1660 cm^‒1^.

Similarly, another peak at 1400 cm^–1^ signalizing CH_2_ wagging of PVDF-HFP and PVP in polymer blend^49^ raised with larger thickness of C + MoO_3_ on wood. The peak at 1280 cm^–1^ on coated wood is assigned to *β* phase of PVDF^50^ and was slightly intensified with larger thickness of C + MoO_3_. An indicative peak on wood coated with C + MoO_3_ at 1190 cm^–1^ is assigned to antisymmetric stretching of CF_2_^51^ and intensifies with amount of C + MoO_3_. The peak at 1050 cm^–1^ as the most intensive on wood indicating the C–O deformation in cellulose, symmetric C–O–C stretching of dialkyl ethers, and aromatic C–H deformations in lignin^52^ completely disappeared with application of C + MoO_3_ on wood. Instead, the peak at 990 cm^–1^, corresponding to the *α*-phase of PVDF–HFP^51^ appeared. Another two peaks at 870 cm^–1^ (indicating *γ* phase of PVDF-HFP) and 840 cm^–1^ (indicating *β* phase of PVDF, –CH_2_– rocking and –CF_2_– stretching) were observed^53^. All these three peaks were promoted with greater film thickness of C + MoO_3_ on wood.

The exposure of uncoated wood to UV light caused a slight increase of the peak at 2930 cm^–1^ (C–H asymmetrical and symmetrical stretching^54^) and a more notable rise of peaks at 1680 cm^–1^ and 1600 cm^–1^, indicating oxidation of lignin^55^ and presence of C–C unsaturated linkages in aromatic rings of lignin^56^. An intensified shoulder peak at 1080 cm^–1^ was attributed to the rearrangements in lignin, specifically the aromatic C–H in plane deformation in guaiacyl units and C–O deformation in primary alcohol^57^. On wood coated with C + MoO_3,_ the peaks at 2930 cm^–1^ and 2850 cm^–1^ diminished with the exposure of coated surfaces to UV light. This indicates the degradation of the remaining cellulose in coated wood by UV light^44^. Moreover, after the exposure to UV light caused the formation of succinimide ((CH_2_)_2_(CO)_2_NH) groups and consequently the main peak at 1680 cm^–1^ remained without a shoulder peak at 1700 cm^–1^. The UV irradiation triggered additional crosslinking reaction and photodegradation of PVP in C + MoO_3_^46,58^.

#### Resistance properties of the coated wood

The results of resistance properties of coated wood surfaces to various impacts are presented in Table 2. The left half of the images shows the assessed surfaces before the resistance property tests, and the right half of the images the appearance of the test surfaces after the completed test. The resistance of the coated wood surfaces to water was rated as good (grade 3), and the damage caused was the same by all three thicknesses of the coating film. The exposure to water probably caused a partial dissolution of the coating since the underlying wood became more visible after the exposure. A similar level of damage was observed by determining the resistance of the surfaces to alcohol (grade 3), while the surface coated with 360 µm was more resistant to alcohol (grade 4). The exposure of the surfaces to the dry heat caused a minor change in colour of the surfaces, and the grade was in all cases 4.

**Table 2:**
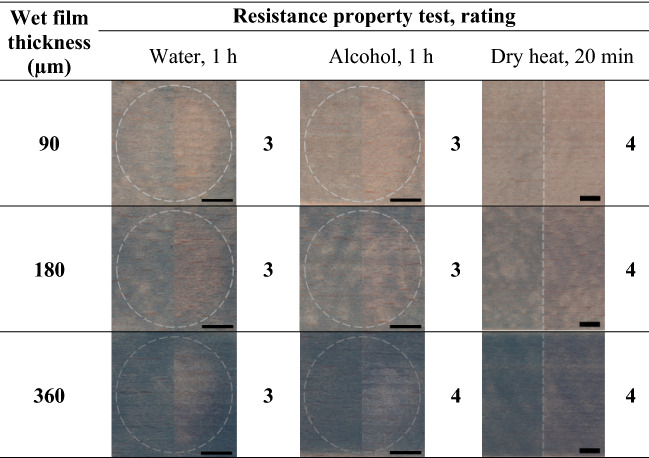
The results of the resistance properties.

The length of the scale bars is 10 mm.

#### Coating adhesion

**Figure 9:**
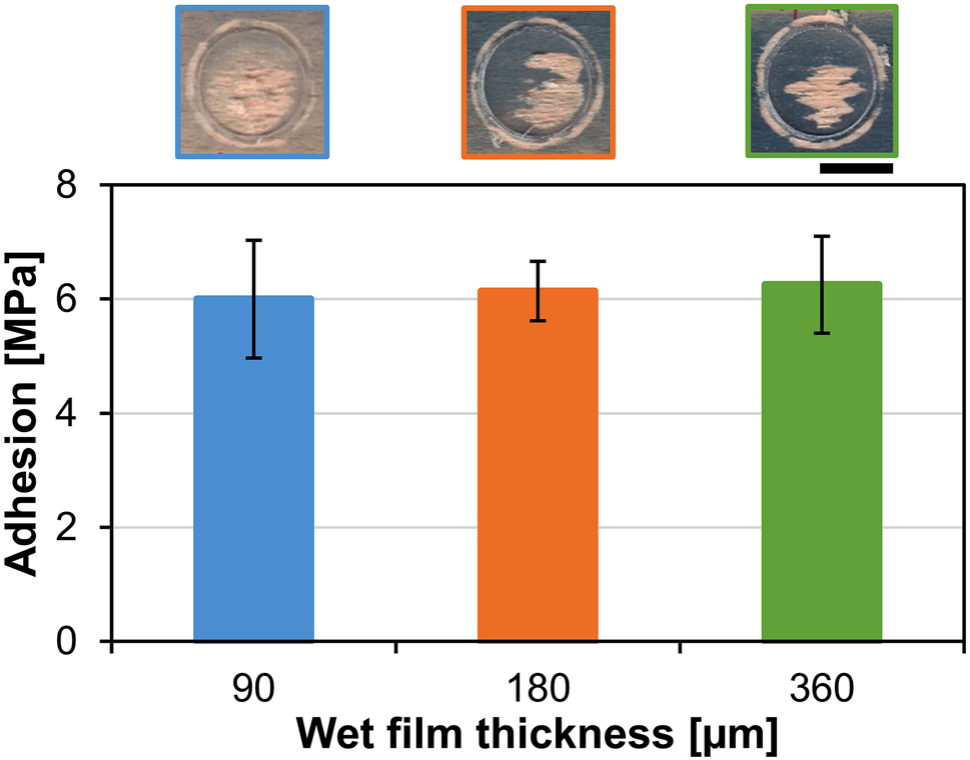
Pull-off adhesion strength of coating films with different wet film thicknesses. The images above the chart show the representative types of failures. The length of the scale bars is 10 mm.

When compared with the common wood coatings reaching the pull-off adhesion strengths between 2 and 3 MPa^59^, the adhesion of the developed C + MoO_3_ coating to wood can be referred as very high, since the values exceeded 6 MPa. Moreover, the adhesion of the coating to the wood substrate was not influenced by the coating film thickness and was generally the same by all of the thicknesses studied (Fig. 9). As seen in the images above the chart in Fig. 9, the adhesion failure between the detached dolly and the coated surface was representative (approximately 60%), while the failure was partially cohesive in the wooden substrate. This indicated that the adhesion of the coating to wood exceeded the cohesive strength of wood and adhesion between the dolly and coated surface.

#### Resistance to blue-staining and moulds

The results of the blue-staining fungal test are presented in the Table 3. The images show the surfaces of exemplary samples from each series before the test start, and surfaces and cross-sections of the same samples after the test was completed in 8 weeks. The given grades of the samples overgrown with blue-stain fungi correspond to the visual assessment with the naked eye and microscopical investigations. The results of the blue-stain fungal test showed that uncoated samples and samples coated with C–MoO_3_ were more overgrown with blue-stain fungi and rated as 3 (average attack). The presence of blue-stain fungi hyphae was observed through the entire cross-section of the samples on both types of samples. The assessment of the samples coated with C + MoO_3_ was more challenging, as the blue colour of the fungal hyphae was slightly hardly distinguishable from the blue surface of the coated wood. However, the blue-staining on C + MoO_3_ samples predominantly appeared on the side surfaces and the edges of the coated surface, while the center of the coated surface remained intact. For these reasons the C + MoO_3_ samples were rated as 2 (slight attack).

**Table 3:**
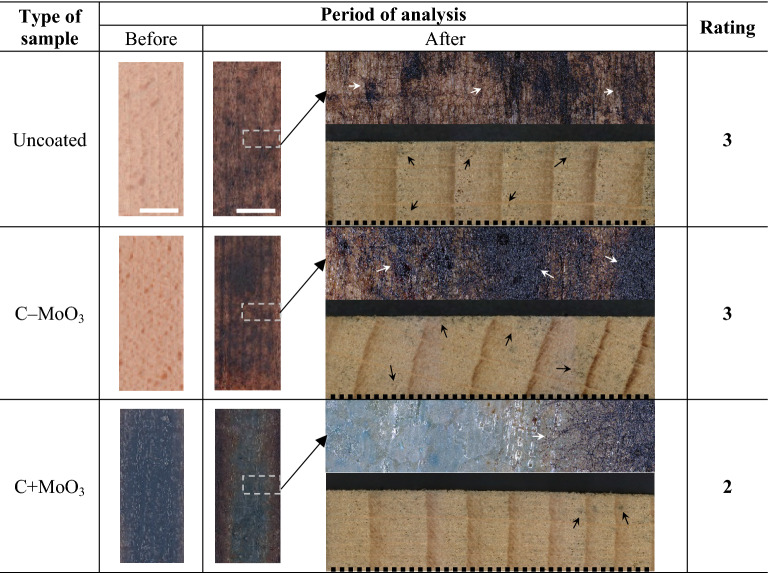
The results of blue-stain fungal test for each type of sample and the corresponding rating.

In the column “After” the upper images show the sample surface and the bottom images show the cross-section of the samples. The open arrows indicate the presence of blue-stain fungi hyphae. The dotted lines indicate an unexposed surfaces. The length of the scale bars is 10 mm.

The results of the resistance to mould growth are presented in Table 4. The images show the surfaces of exemplary samples from each series before the start of the test and the images of the same surfaces during 8 weeks of exposure to mould growth in humid environment. As detected with the naked eye, the mould appeared on all three types of samples after completed 7 weeks of exposure. Due to the darker colour of C + MoO_3_, the light-shaded mould was especially visible on samples coated with C + MoO_3_. However, darker conidiophores were detected only on the uncoated samples and samples coated with C–MoO_3_, but not on samples coated with C + MoO_3_. At the end of exposure, the presence of well-developed conidiophores was confirmed with the microscope on the uncoated sample, giving these the grade 4. On the samples coated with C–MoO_3,_ the conidiophores seemed to be at the beginning of development (grade 3). On the samples coated with C + MoO_3_ no development of conidiophores was detected after the completed test, and the grade 0 was given to this set of samples.

**Table 4:**
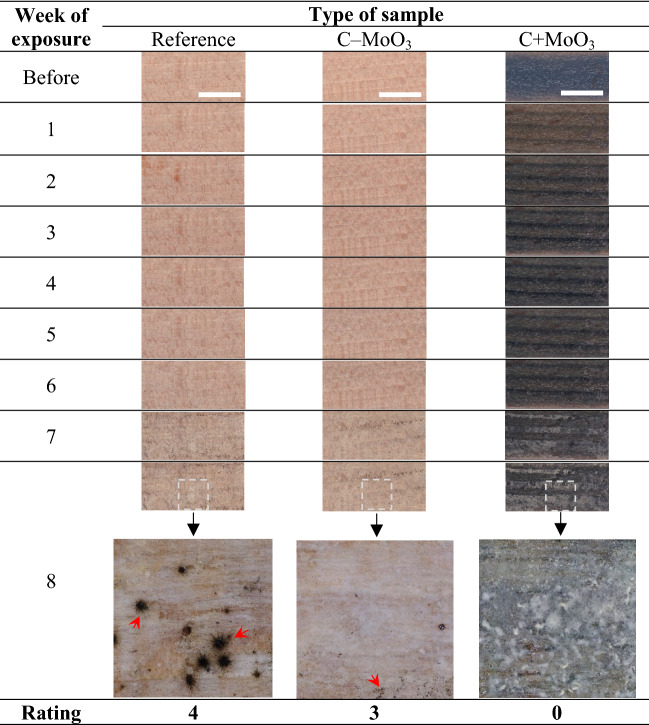
The results of the resistance to mould growth test for each type of sample and the corresponding rating.

The red open arrows indicate the presence of mould conidiophores in wood. The length of the scale bars is 10 mm.

#### Comparison of the performance

To get a better overview of the properties of C + MoO_3_, these are compared in Table 5 with the properties of the commercially available interior coatings whose properties were reported in the listed references. It should be mentioned that many of these parameters are defined by the properties of the coating itself (e.g. type of binder), the properties of the coating system (e.g. number of layers) or the properties of the surface system, which includes also the properties of the substrate (e.g. type of wood). The properties of C + MoO_3_ were in some respects more and in some respects less comparable to the values from the other studies. For example, the hardness of C + MoO_3_ was even up to 2-times higher than usually due to the thinner layers. The same is true for the roughness of the coated wood surface; a greater dry film thickness of the coating smooths the surface of the wood substrate more effectively. The colour changes caused by artificial weathering additionally depend on the weathering parameters (temperature and humidity conditions, type of light source, exposure time, etc.), so it is difficult to compare the ΔE* values with those obtained in other studies. Once again, good performance was confirmed in terms of C + MoO_3_ adhesion and mould resistance of wood coated with C + MoO_3_.

**Table 5:** The overview of results gained in this study and their comparison with the references.

Property	Compared materials				
	C + MoO_3_	Comparative material			
		Value	Coating type	Substrate	Reference
Non-volatile content (%)	21	17‒29	Waterborne and polyurethane coating	–	^60^
Surface tension mN × m^–1^	33.2	50.9	Waterborne coating	–	^61^
Coating film hardness (s)	96‒126	43‒80	Acrylate and polyurethane systems	–	^35^
CAs of coating on wood (°)	26	33	Waterborne coating	Norway spruce (*Picea abies* (L.) Karst.) and beech	^61^
Surface roughness (µm)	10.6‒16.2	2.6‒3.7	Waterborne and polyurethane coating	Oriental beech (*Fagus orientalis* L.)	^62^
Dry film thickness (µm)	3‒40	51	Polyurethane coating	Indian rosewood (*Dalbergia sissoo* Roxb.)	^63^
CAs of water on coated wood (°)	98‒123	122	Waterborne acrylic coating	Chinese white pine (*Pinus armandii* Franch.)	^64^
Colour changes	4.8‒14.4	18‒21	Solventborne and waterborne coatings	Norway spruce (*Picea abies* (L.) Karst.) and beech	^65^
Resistance against water and heat	3 and 4	4 and 5	Acrylate and polyurethane systems	European oak (*Quercus* sp.)	^35^
Coating adhesion (MPa)	6	3‒3.5	Acrylic and polyurethane coating	Oriental beech (*Fagus orientalis* L.), Scots pine (*Pinus sylvestris* L.), and cherry (*Prunus cerasus* L.)	^66^
Resistance to moulds	0	3‒4	Polyurethane and polyurethane-acrylate coatings	Beech	^67^

## Conclusions

A novel PVDF-HFP/PVP polymer composite containing MoO_3_ nanoparticles with the ability to form coating films was successfully synthesized. At first, the experiments presented in the article showed that the hardness of the C + MoO_3_ films depended on the coating film thickness (tested wet film thicknesses 90 µm, 180 µm and 360 µm). The developed coating showed the ability to form the surface system with the surface of beech wood. C + MoO_3_ expressed good wetting abilities on the wood surface, reaching the initial values of CAs of about 15.6° together with fast spilling on the wood surface. The roughness of coated wood surface increased with the larger thickness of C + MoO_3_ film, due to the etching of the wood surface by the dimethylformamide solvent in the coating. The C + MoO_3_ well penetrated in the wood surface microstructure, forming the cured coating films with thickness from only 3 µm (applied 90 µm) to 40 µm (applied 360 µm). The presence of C + MoO_3_ on a wood surface made it considerably more hydrophobic when compared to uncoated wood, reaching the values of water CAs up to about 123°. The irradiation of wood surface coated with C + MoO_3_ with UV light caused visible colour changes on the tested samples, which was together with oxidation of lignin and degradation of cellulose in wood, a consequence of the photodegradation of PVP in C + MoO_3_ coating. The coated wood surfaces exhibited good resistance to water, alcohol and dry heat (grade 3–4), while the adhesion of the C + MoO_3_ to the wood surface was excellent (6 MPa), regardless of the coating film thickness. The antimicrobial testing showed that the presence of MoO_3_ in the developed coating played an important role by the resistance to blue-stain fungi and mould development. While the uncoated wood samples and samples coated with C–MoO_3_ experienced an average attack (grade 3), the samples coated with C + MoO_3_ experienced a slight attack (grade 2) from the blue-stain fungi. The contribution of MoO_3_ presence was even more evident by testing the resistance of the samples to mould growth. After 7 weeks of testing, the conidiophores were well developed on the uncoated samples (grade 4), slightly less on samples coated with C–MoO_3_ (grade 3), but absent on the samples coated with C + MoO_3_ (grade 0).

In conclusion, the developed PVDF-HFP/PVP polymer composite with MoO_3_ nanoparticles has a good ability to interact with wood surfaces and can potentially be used as a protective coating for wood, in terms of protection to physical impacts as well as to protect the wood from microbes.

## Data Availability

The datasets used and/or analysed during the current study available from the corresponding author on reasonable request.
